# Improving Prediction of Favourable Outcome After 6 Months in Patients with Severe Traumatic Brain Injury Using Physiological Cerebral Parameters in a Multivariable Logistic Regression Model

**DOI:** 10.1007/s12028-020-00930-6

**Published:** 2020-02-13

**Authors:** Frank C. Bennis, Bibi Teeuwen, Frederick A. Zeiler, Jan Willem Elting, Joukje van der Naalt, Pietro Bonizzi, Tammo Delhaas, Marcel J. Aries

**Affiliations:** 1 Department of Biomedical Engineering, Maastricht University, PO Box 616, 6200 MD Maastricht, The Netherlands; 2 MHeNS School for Mental Health and Neuroscience, Maastricht University, PO Box 616, 6200 MD Maastricht, The Netherlands; 3 CARIM School for Cardiovascular Diseases, Maastricht University, PO Box 616, 6200 MD Maastricht, The Netherlands; 4 Section of Neurosurgery, Department of Surgery, Rady Faculty of Health Sciences, University of Manitoba, Winnipeg, Canada; 5 Department of Human Anatomy and Cell Science, Rady Faculty of Health Sciences, University of Manitoba, Winnipeg, Canada; 6 Biomedical Engineering, Faculty of Engineering, University of Manitoba, Winnipeg, Canada; 7 Division of Anaesthesia, Department of Medicine, Addenbrooke’s Hospital, University of Cambridge, Cambridge, UK; 8 Department of Clinical Neurophysiology, University of Groningen, University Medical Center Groningen, Groningen, The Netherlands; 9 Department of Neurology, University of Groningen, University Medical Center Groningen, Groningen, The Netherlands; 10 Department of Data Science and Knowledge Engineering, Maastricht University, Maastricht, The Netherlands; 11 Department of Intensive Care, Maastricht University Medical Center, Maastricht University, Maastricht, The Netherlands

**Keywords:** Traumatic brain injury, Neuromonitoring, Outcome, Prediction, Logistic regression

## Abstract

**Background/Objective:**

Current severe traumatic brain injury (TBI) outcome prediction models calculate the chance of unfavourable outcome after 6 months based on parameters measured at admission. We aimed to improve current models with the addition of continuously measured neuromonitoring data within the first 24 h after intensive care unit neuromonitoring.

**Methods:**

Forty-five severe TBI patients with intracranial pressure/cerebral perfusion pressure monitoring from two teaching hospitals covering the period May 2012 to January 2019 were analysed. Fourteen high-frequency physiological parameters were selected over multiple time periods after the start of neuromonitoring (0–6 h, 0–12 h, 0–18 h, 0–24 h). Besides systemic physiological parameters and extended Corticosteroid Randomisation after Significant Head Injury (CRASH) score, we added estimates of (dynamic) cerebral volume, cerebral compliance and cerebrovascular pressure reactivity indices to the model. A logistic regression model was trained for each time period on selected parameters to predict outcome after 6 months. The parameters were selected using forward feature selection. Each model was validated by leave-one-out cross-validation.

**Results:**

A logistic regression model using CRASH as the sole parameter resulted in an area under the curve (AUC) of 0.76. For each time period, an increased AUC was found using up to 5 additional parameters. The highest AUC (0.90) was found for the 0–6 h period using 5 parameters that describe mean arterial blood pressure and physiological cerebral indices.

**Conclusions:**

Current TBI outcome prediction models can be improved by the addition of neuromonitoring bedside parameters measured continuously within the first 24 h after the start of neuromonitoring. As these factors might be modifiable by treatment during the admission, testing in a larger (multicenter) data set is warranted.

**Electronic supplementary material:**

The online version of this article (10.1007/s12028-020-00930-6) contains supplementary material, which is available to authorized users.

## Introduction

Severe traumatic brain injury (TBI) is defined as severe trauma to the brain and skull due to an external force. In Europe, 57.000 TBI-related deaths are reported each year [1]. TBI is the leading cause of death and severe disability in young adults [2]. The external force to the brain may result in ischaemia, contusions and haematomas. These processes lead to swelling, rise in intracranial pressure (ICP), decrease in cerebral perfusion pressure (CPP) and finally cerebral ischaemia [2]. Intensive care unit (ICU) admission with organ support is necessary in comatose TBI patients to overcome secondary damage. Severe or moderate disability is common in surviving patients, which makes TBI a large burden for patients, families and society [3]. An accurate prediction of outcome would be helpful, as it would support the clinical team in decision-making and discussions with the family during ICU admission.

Models such as the (extended) model based on data from the Corticosteroid Randomisation after Significant Head Injury (CRASH) study are developed to predict the 6-month individual outcome [4, 5]. These models primarily use baseline demographics and factors related to the primary injury to predict outcome. However, confounding factors, consequences of the initial trauma (like brain swelling, metabolic crises and inflammation) and the individual response to therapy during ICU admission are not included. Because full supportive care for a certain amount of time from initial presentation is recommended to maximize the potential for recovery from primary and secondary damage [6], extending prognostic models with early physiological monitoring data might improve the outcome prediction accuracy as has been shown in studies on the ICU for pathologies other than TBI [7–9].

Commonly used parameters for continuous hemodynamic monitoring in the ICU are heart rate (HR) and mean arterial blood pressure (MAP). For cerebral monitoring, the guidelines recommend ICP and CPP monitoring [10, 11]. These parameters depend heavily on the treatment given and are associated with mortality [12, 13] but are limited in their correlation with unfavourable outcome. Therefore, the use of additional cerebral parameters such as the cerebral compliance and autoregulation has been suggested for therapy guidance [14]. Dynamic cerebral autoregulation parameters such as the cerebrovascular pressure reactivity index (PRx) are gaining more interest because these parameters are correlated independently with TBI outcome [14–17].

In this retrospective study, we aim to develop a model that combines the prediction of the CRASH model with continuously measured general and brain-specific monitoring parameters in severe TBI patients on day one after the start of neuromonitoring. We hypothesize that extending the prediction model improves outcome prediction and may assist decision-making during the ICU stay.

## Materials and Methods

### Design and Subjects

Patients from the ICU of the University Medical Centre Groningen (UMCG) and Maastricht University Medical Centre (MUMC), both in the Netherlands, were retrospectively analysed. Data were included from two centres to increase the number of patients. In both the centres, medical ethical committees approved anonymized physiological, diagnostic, clinical and outcome data collection. The need for informed consent was waived in Groningen. In Maastricht, informed consent was obtained from the closest relative. Inclusion occurred between May 2012 and March 2015 in the UMCG and between April 2017 and January 2019 in the MUMC. Inclusion criteria were (1) severe TBI and (2) ICP/CPP monitoring. Exclusion criteria were (1) moribund at admission, (2) pregnancy, (3) monitoring started > 24 h after trauma, (4) loss to follow-up or (5) incomplete baseline data for the extended CRASH score.

### Data Collection

Outcome after 6 months was obtained by consultation over the phone by a clinician. The outcome of patients was scored on the five-point Glasgow Outcome Scale (GOS). The GOS was subsequently dichotomized to unfavourable (dead, vegetative state or severe disability [GOS 1–3]) or favourable (mild disability or full recovery [GOS 4–5]) outcome. Mortality after 14 days was not evaluated as this was not standardly collected. The patients were treated by the same neuro-intensivist in both hospitals (MJ Aries).

Electrocardiogram, arterial blood pressure (ABP) and ICP were recorded at 250 Hz using ICM + software (www.icmplus.neurosurg.cam.ac.uk) running on a bedside computer. In Groningen, an external ventricular drain was used with an electronic (ICP) sensor in the tip for intraventricular pressure monitoring and optional cerebral spinal fluid drainage capacity (Neurovent, RAUMEDIC AG, Helmbrechts, Germany). In Maastricht, a parenchymal sensor was used. ABP was zeroed at heart level in Groningen. In Maastricht, ABP was zeroed at heart level up to 2018, after which ABP was measured at the brain level. Corresponding HR, ABP and ICP were down-sampled to 1 sample/min. PRx was calculated as the moving Pearson correlation of 30 consecutive 10-s non-overlapping moving window averages of ABP and ICP, updated every minute, resulting in one averaged correlation value per minute. The PRx index indicates the intactness of the cerebrovascular reactivity. With an intact cerebrovascular reactivity, a slow increase in ABP will be counteracted by cerebral vasoconstriction, leading to a decrease in cerebral volume and subsequent in ICP. If the cerebrovascular reactivity is impaired, the cerebral volume and ICP will rise as a result of an increase in ABP. Therefore, the correlation coefficient will be negative or around zero in the case of an intact cerebrovascular reactivity, and positive when the cerebrovascular reactivity is impaired. Similar correlation-based parameters calculate the correlation coefficient in the same way, but by using different input and output parameters. These correlation-based parameters are the correlation coefficient between: ABP and pulse amplitude of ICP (AMP) (pressure amplitude index [PAx]), which describes the cerebrovascular reactivity; moving correlation coefficient between AMP and CPP (RAC), which includes information about the cerebral compensatory reserve and cerebrovascular reactivity; moving correlation coefficient between AMP and ICP (RAP), which describes the intracranial compliance. For more in-depth information about these dynamic parameters, we refer to recent literature [16, 18]. The extended CRASH risk score was calculated using basic clinical parameters obtained at admission [4].

### Physiological Parameters and Methodology

MATLAB (2018A, The MathWorks, Natick, MA, USA) was used for all analyses. Outliers were replaced in HR, MAP and ICP with the *filloutliers* function. The algorithm replaced data points more than six median absolute deviations above or below the median by a linearly interpolated value using the previous and the next data point. No outliers were removed in parameters describing the trends in cerebral compliance, compensatory reserve and autoregulation, as individual data points are known to be noisy [19].

We investigated which of four data periods after the start of neuromonitoring were most informative to predict outcome. These periods contained data from 0–6 h, 0–12 h, 0–18 h and 0–24 h after the start of monitoring. Missing data were not replaced. If a period contained less data than 50% of the total length or less than 3 h additional to the previous period, the period was excluded. We chose to exclude only time periods and not the entire patient, in order to represent the clinical setting. In total, 15 parameters for each period were selected representing different physiological domains: (1) average ICP, ABP and HR; (2) average PRx, PAx, RAP and RAC; (3) the slope of a linear line fitted on the PRx, PAx and RAC (as an indicator how these parameters progress over time); (4) the amount of impairment of autoregulatory parameters, defined as the area of the signal above a set threshold divided by the total amount of samples: for the PRx (threshold  > 0.35), PAx (> 0.25), RAC (> − 0.05) and ICP ( > 2 mmHg) [12]; and (5) the individual unfavourable outcome risk score (CRASH, %). This resulted in 15 values per time segment.

### Logistic Regression Modelling

**Fig. 1:**
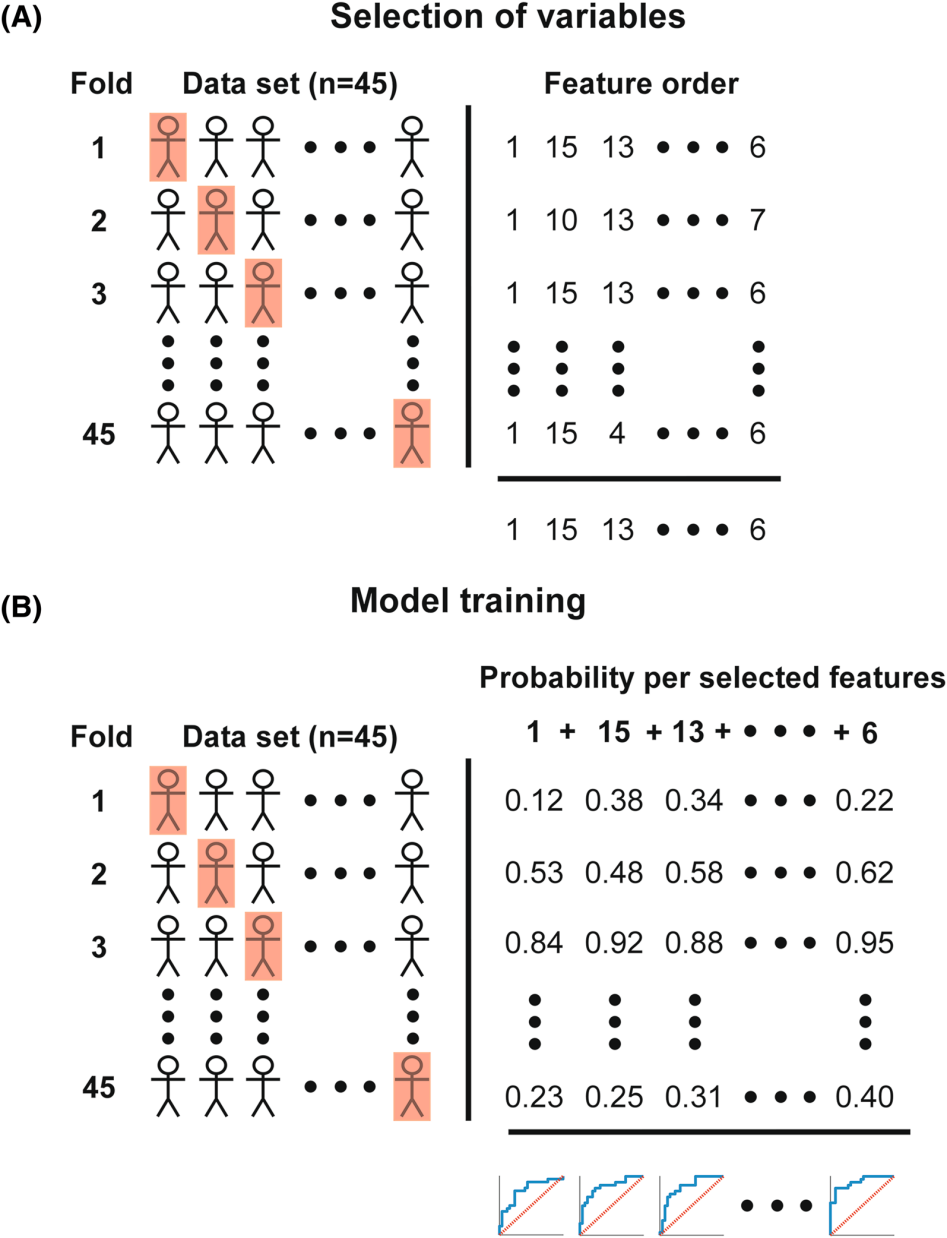
**a** Selection of the ideal order of parameters for the selected time period (for instance 0–6 h) using leave-one-out cross-validation and forward feature selection. The data set selects a different test set (red square) for each fold. Parameters are ordered subsequently for each fold. The parameters which are seen most often for each column are selected as the order for the final model. **b** Model training using the previous selected parameters, each time adding the next best parameter. The data again select a different test set for each fold. A logistic regression model is trained and tested for each fold, resulting in the probability of an unfavourable outcome for each subject. An ROC curve is created using this probability for all subjects over a single number of features included. The ROC with the best AUC is selected as the final model for this time segment. *AUC* area under the curve, *ROC* receiver operating characteristic (Color figure online)

A logistic regression outcome model was trained based on the above parameters, called the ‘combined’ model. To train the model with those parameters that are most predictive of outcome, the algorithm will first rank the parameters on their respective predictive power (Fig. 1).

### Ranking of Parameters

To rank the parameters, leave-one-out cross-validation (LOOCV) [20] was combined with forward feature selection (FFS) (Fig. 1a) [21]. LOOCV splits the data set 45 times (called folds) in a training set containing all but one and a test set containing the remaining patient. For each fold, the training set was used to determine the optimal order of parameters by FFS, from best to worst. The CRASH risk score was fixed to be the first ranked parameter. This results in an optimal order of parameters for each patient when not including that patient in the FFS. To define the overall optimal order of parameters, the kth optimal parameter is selected as the most occurring parameter at position k from all 45 LOOCV models. This is continued until all parameters are included, resulting in an overall ranking of the 15 parameters.

### Training the Model

To train the model, the data set is again divided by LOOCV (Fig. 1b) and training and testing are performed for each fold. Starting with the neuromonitoring-derived parameter that was ranked highest as single input, a logistic regression model is trained on the training set and tested on the test set for each split. The output of the test set is the probability that the patient has an unfavourable outcome. As this is repeated for each fold, the probability of an unfavourable outcome is predicted for each patient. Thereafter, a model that included the two highest ranked parameters was trained and tested, again resulting in an individual probability of unfavourable outcome. This is repeated until all parameters are added. The number of parameters with the highest area under the curve (AUC) in the corresponding ROC curve is selected as the best model for that time period. A common problem in machine learning is overfitting, which occurs when the number of parameters is too large compared to the sample size. Because the number of patients is limited, a maximum of six parameters were included. The selection of parameters and training of the model was performed for each time period.

The accuracy of the model, expressed in correctly predicted patients, is based on the optimal cut-off value determined by the Youden’s index [22]. The performance of the CRASH model and the different combined models will be compared using the ROC curves, the AUC values and the prediction accuracy. We decided not to statistically compare AUC curves due to the limited sample size, and hence, results should be interpreted at a qualitative level. Model calibration is assessed by visualization of the predicted probabilities versus the actual outcome.

## Results

Sixty-two patients were eligible for inclusion. Two patients did not have any data. Eleven patients were excluded because monitoring started > 24 h after the trauma. The outcome was unknown in four of the remaining 49 patients. In total, 45 patients were available for analysis.

The patients had a median age of 41 years (interquartile range [IQR] 24–57) and a median admission Glasgow Coma Scale (GCS) of 7 (IQR 5–9). Thirty-three patients were male (73.3%). In 11 patients, ABP was zeroed at the brain level. In three patients, a secondary decompressive craniectomy was performed. In 29 out of 45 patients, the mechanism of injury involved road traffic accidents. In 13 patients, the mechanism of injury was a fall of height. The remaining three patients involved an assault, metal against head and a hit by a tree. The patients had a median Marshall computed tomography score of 2, with the 25 and 75 percentile also at 2. Four patients had evacuated mass lesions. Dividing patients according to the GOS, 13 (28.9%) died, 1 (2.2%) was in a vegetative state, 6 (13.3%) experienced severe disabilities, 10 (22.2%) had moderate disabilities and 15 (33.3%) made a good recovery. Twenty patients (44.4%) had an unfavourable outcome. All 45 patients were included in the 0–6 h and 0–12 h group. We excluded 1 patient (GOS score 5) in the 0–18 h group, whereas we had to exclude 4 patients (GOS score 4–5) in the 0–24 h group because data collection was stopped due to early removal of the ICP monitor as a result of low ICP values. The mean parameter values are given in Supplemental Material 1, Table S1.

### Performance of Different Models

**Fig. 2:**
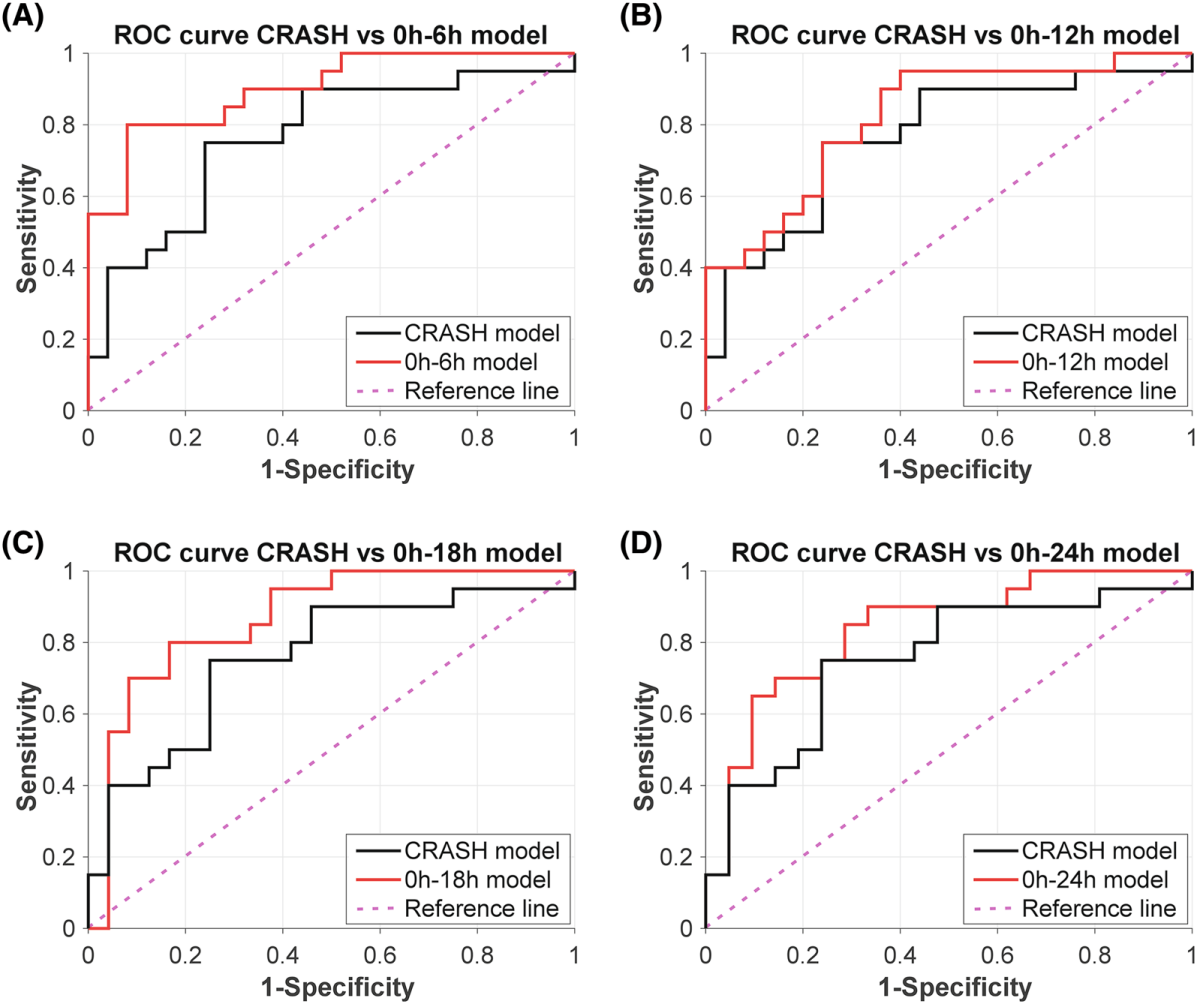
Models predicting unfavourable outcome in severe TBI patients and ICU admission. ROC curves of the CRASH model and the combined model for each time period. High AUC values are seen for the logistic model predicting unfavourable outcome, especially for the early monitoring period (**a**).* AUC* area under the curve,* CRASH*  Corticosteroid Randomisation After Significant Head Injury,* ICU* intensive care unit,* ROC*  receiver operating characteristic,* TBI * traumatic brain injury

**Fig. 3:**
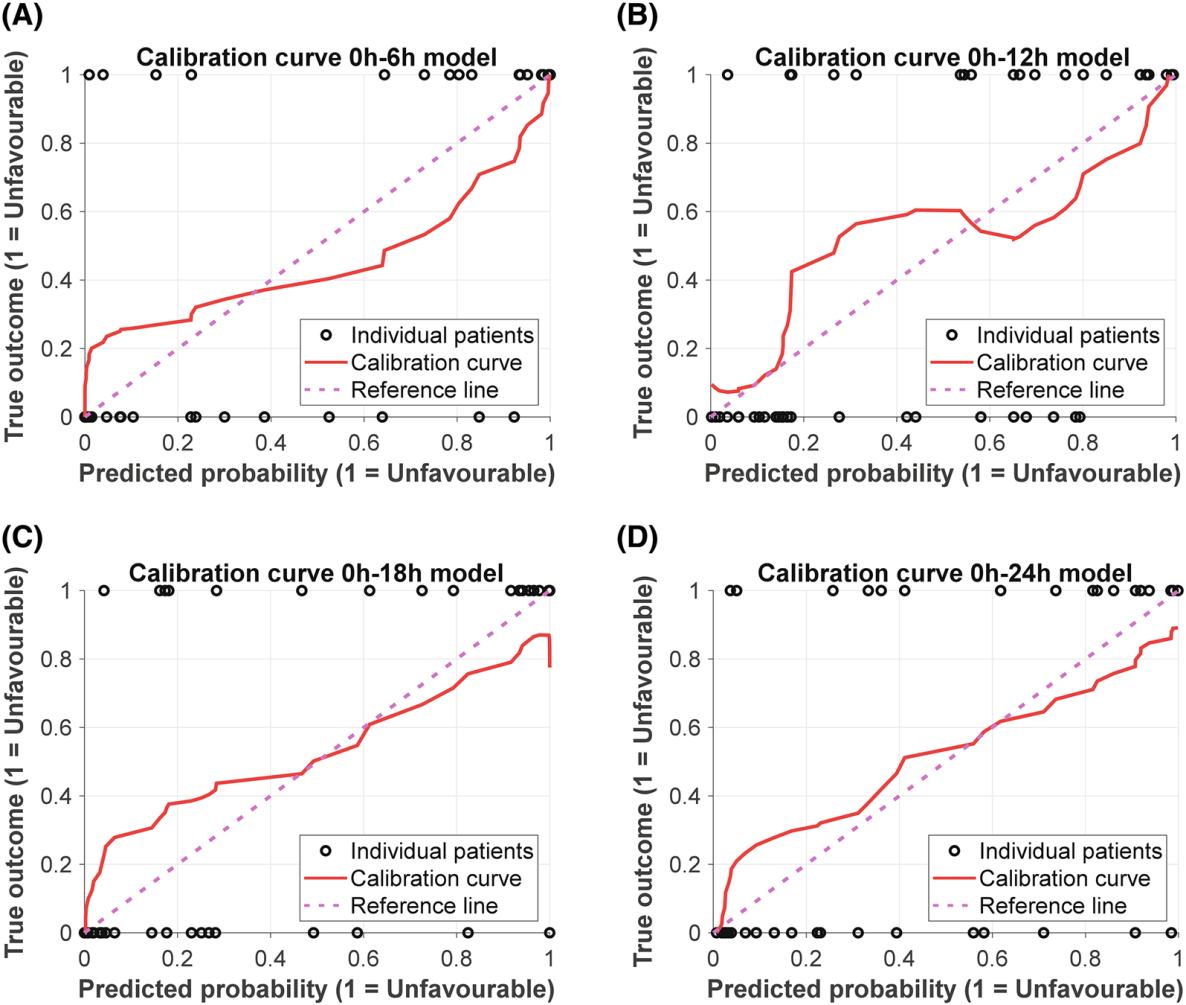
Calibration plots of the combined models predicting unfavourable outcome in severe traumatic brain injury patients. A systematic underestimation is seen for the lower predicted probabilities, whilst a systematic overestimation is seen for higher predicted probabilities

The CRASH model performs adequate for all periods (AUC 0.75–0.76, accuracy 75.0–75.6%) (Table 1, Fig. 2). Slight deviations in CRASH performance are due to the fact that not all time periods had an equal number of subjects. The combined model shows a high AUC and accuracy for each time period, especially in the early monitoring period (0–6 h, AUC 0.90, accuracy 86.7%). The parameters included per time segment are shown in Table 2. The logistic regression model coefficients and thresholds for favourable vs unfavourable prediction are given in Supplemental Material 2, Table S2–S5. The calibration curves indicate a systematic underestimation for the lower predicted probabilities, whilst a systematic overestimation for the higher predicted probabilities is observed (Fig. 3). However, as the data set used in this study is fairly small and the magnitude of error is not severe, calibration is deemed acceptable [23].

**Table 1:** Models predicting unfavourable outcome in severe TBI patient and ICU admission

Time period	CRASH risk score		Combined models	
	AUC (CI)	Prediction accuracy (%)	AUC (CI)	Prediction accuracy (%)
0–6 h (*n* = 45)	0.76 (0.62–0.91)	75.6	0.9 (0.8–1)	86.7
0–12 h (*n* = 45)	0.76 (0.62–0.91)	75.6	0.82 (0.69–0.95)	75.6
0–18 h (*n* = 44)	0.76 (0.61–0.9)	75.0	0.87 (0.76–0.98)	81.8
0–24 h (*n* = 41)	0.75 (0.6–0.9)	75.6	0.84 (0.71–0.96)	78.0

The AUC and prediction accuracy for the best model using the CRASH and the combined model for each time period. Higher AUC values and prediction accuracies can be seen for the combined model for predicting unfavourable outcome after severe TBI and ICU admission

*AUC* area under the curve, *CI* confidence interval, *CRASH* Corticosteroid Randomisation After Significant Head Injury, *ICU* intensive care unit, *TBI* traumatic brain injury

**Table 2:** Parameters included per time segment

^Time segment^	Parameter included					
	First	Second	Third	Fourth	Fifth	Sixth
CRASH	CRASH					
0–6 h	CRASH	Mean ABP	Slope PAx	Slope PRx	Slope RAC	
0–12 h	CRASH	Impairment PRx	Mean ABP			
0–18 h	CRASH	Mean PRx	Mean PAx	Mean ABP	Impairment RAC	Slope RAC
0–24 h	CRASH	Impairment PRx	Slope PAx	Slope PRx	Mean ABP	

Ordering is the sequence in which forward feature selection picked the parameters

*ABP* arterial blood pressure, *CRASH* Corticosteroid Randomisation After Significant Head Injury, *PAx* Pearson correlation coefficient between ABP and pulse amplitude of the intracranial pressure, *PRx* Pearson correlation coefficient between ABP and intracranial pressure, *RAC* Pearson correlation coefficient between pulse amplitude of the intracranial pressure and cerebral perfusion pressure. Parameters were included per time segment in the combined model

### False Classification

Both the CRASH and the combined models classified a few more patients to have a favourable than an unfavourable outcome (Table 3). The number of misclassified subjects was comparable over all GOS outcomes. The percentage of patients who were classified to have a favourable outcome but actually died ranged from 7.7% (1 out of 13) to 46.2% (6 out of 13). The percentage of patients who were predicted to have an unfavourable outcome but actually had a GOS score of 5 ranged from 0 to 26.7% (4 out of 15). The combined model using data of 0–6 h showed the highest AUC, the least mortality misclassifications and the second-best good recovery misclassifications.

**Table 3:** True GOS scores for misclassifications per model

^Time segment^	^True GOS score^					
	1	2	3	4	5	Total
CRASH	2/13	1/1	2/6	3/10	3/15	11/45
0–6 h	1/13	0/1	3/6	0/10	2/15	6/45
0–12 h	3/13	0/1	2/6	2/10	4/15	11/45
0–18 h	2/13	0/1	2/6	2/10	2/14	8/44
0–24 h	6/13	0/1	1/6	2/8	0/13	9/41

Number of false favourable (true GOS score of 1, 2, 3) and false unfavourable predictions (true GOS score of 4, 5) vs total number of subjects per true GOS score

*CRASH* Corticosteroid Randomisation After Significant Head Injury, *GOS* Glasgow Outcome Scale

## Discussion

This pilot study aimed to develop a model that combined baseline parameters with continuously measured general and brain-specific monitoring parameters in admitted severe TBI patients to improve the prediction accuracy for the six-month clinical outcome. The combined models showed high AUC values when using data from 0–6 h up to 0–24 h period, with the highest performance when using data of the first 6 h (Fig. 2, Table 1). Furthermore, an increase in prediction accuracy was found in 3 out of 4 time periods, with the best performance when neuromonitoring data of the first 6 h were used. Our results are in line with our hypothesis that current TBI outcome prediction models can be improved by the addition of early neuromonitoring data.

Of special interest are the parameters selected by the model. Mean ABP was included for each time period, with lower ABP resulting in a higher chance of unfavourable outcome. This is in line with current clinical practice, as ABP is part of all treatment protocols for critically ill patients. Lower systolic blood pressures before and during ICU admission are related to higher mortality rates in TBI patients [13, 24]. The finding that ABP was selected in every model—despite the fact that different ABP zeroing levels were applied in patients—is interesting because autoregulation-based parameters are more likely to be included by the model due to the fact that they do not rely on the absolute level of ABP. Additional analyses of models including CRASH and mean ABP as sole parameters show an AUC and prediction accuracy of 0.82 and 77.8%, 0.79 and 75.6%, 0.76 and 72.7% and 0.74 and 75.6% for the 0–6 h, 0–12 h, 0–18 h and 0–24 h time spans, respectively. These findings indicate that particular combinations of physiological derangements may be (prognostically) important (for example the combination of low ABP in a situation with impaired autoregulation). Subsequent parameters were all correlation-based parameters describing trends in cerebrovascular (autoregulation) reactivity or cerebral compensatory reserve. Of these correlation-based parameters, different characteristics were selected (slope, mean and amount of impairment). These findings indicate that not only a general ICU parameter (ABP) but also specific parameters representing the unique (global) cerebral homoeostasis and protection mechanisms contain prognostic information. We speculate that trends in these parameters can be used in addition to baseline prognostic parameters to aid decision-making and discussion between the clinical team and families during the ICU admission period.

The finding that the effect of including neuromonitoring data in a prediction model for 6-month outcome is the largest when using data from the ‘early’ 0–6 h time period may be because we studied trauma patients in which treatment was focused on controlling ICP as the main goal. These treatment effects may have reduced the effect of the selected parameters in the combined model. However, in the early phase of admission, the patient may not yet be fully stabilized and thus the parameters may reflect the deranged physiological status of the patient better. Including treatment intensity level may account for treatment given in a later stage. The treatment effect may be most apparent in the fact that ICP was never selected, as current therapy protocols are primarily focused on controlling ICP. Recent literature has shown that cerebrovascular reactivity/autoregulation parameters, however, appear to remain relatively independent of the ICP-guided treatment [25–27]. As cerebrovascular reactivity or autoregulation status might be modifiable by directed (perfusion) therapies started as early as possible, our findings might add retrospective evidence for the call for prospective testing.

### Limitations

This study has several limitations. First, the sample size of this study is small. Therefore, we did not have the possibility to use a separate test set. Instead, we performed feature selection in a separate cross-validation before training the model. Selection of parameters by FFS may vary between subjects in the case of correlation between parameters, as the information between parameters is quite similar. As some parameters are correlated in this study, the order of parameters in different patients as selected by FFS varied. Therefore, variability in feature selection and subsequent low performance would be seen if FFS was performed in the cross-validation splits together with prediction. Although the current method might introduce a slight bias, it ensures that each model uses the same features. To improve training and ensure correct generalization, we recommend increasing the sample size and validate the found models on an external data set. Second, the parameters used show correlation, especially parameters describing autoregulation such as PRx and PAx. The feature selection algorithm used in this method does not take correlation into account. Although this is partly solved due to the selection of parameters that perform best most often, it is recommended to use algorithms that are capable of handling correlation in parameters, such as lasso logistic regression. To test the influence of correlation between parameters, model performance was evaluated once without PRx and once without PAx. AUC was the highest using both PRx and PAx in three out of the four time segments and equal in the remaining one time segment (data not shown). Therefore, although the correlation is present, including both parameters improves prediction accuracy. We hypothesize that both might contain different cerebral hemodynamic information. Third, selection of the 14 monitoring-based parameters was based on availability and proven relationship to outcome in the literature. The parameters in this study are mainly perfusion related, whilst brain oxygenation or metabolism is not considered. Adding the latter to the model, for instance using near-infrared spectroscopy or parenchymal brain tissue oxygenation, may further increase the predictive value. Fourth, it is possible that events occur after the first 24 h, such as a deterioration or improvement in the measured physiological parameters, complications or independent issues, such as unrelated death after discharge. Future models should consider incorporating such long-term deviations in addition to the early-phase parameters. Fifth, the combined model included ICU-admitted severe TBI patients, whilst the CRASH model was trained on all TBI patients with a GCS lower than 14 [4]. Therefore, the CRASH model is not optimized for our specific data set. If the initial CRASH model accuracy would be higher, the addition of early neuromonitoring data may result in even higher prediction accuracies than currently found. In future studies, we will include the model created on the International Mission for Prognosis and Analysis of Clinical Trials (IMPACT score) in TBI database [5], which may result in additional info and thus better outcome prediction. Sixth, the model did not directly take the influence of treatment into account. In future work, the (intracranial hypertension) treatment intensity level is worth adding as a separate parameter to the model. Last, this study used the time from the start of neuromonitoring to divide data in time segments. However, the start of neuromonitoring (data collection) may be postponed due to for example operator availability or delayed ICU arrival due to urgent (life-saving) surgery. Dividing data according to time after trauma and adding parameters describing time and procedures from trauma to ICU admission/data collection may improve outcome prediction.

Prognostication in ICU patients can be improved by physiological parameters. Meiring et al. [7] showed that common physiological parameters such as HR and MAP and treatment given can predict mortality on the ICU on subsequent days. Other applications are prediction of delayed cerebral ischaemia after subarachnoid haemorrhage [9], prediction of favourable neurological outcome among children on the ICU with critical illness [8] or prediction of impending sepsis in neonates [28]. Although it also has been attempted to use physiological parameters to predict outcome 6 to 12 months after TBI, data used are solely measured before admission or incorporate the whole ICU admission period, hampering (early) clinical assistance [4, 5, 29–31].

## Conclusions

This study showed that the inclusion of (complex) physiological data of the first 24 h after admission improves the prediction of the 6-month outcome in TBI patients. The main perfusion-related parameters included are ABP and parameters describing cerebral compliance and autoregulation. As these parameters might be modifiable by treatment during the admission, testing in a larger (multicenter) data set is warranted.

## Electronic supplementary material

Below is the link to the electronic supplementary material.

Supplemental material 1: Mean physiological value for each parameter per time segment (DOCX 14 kb)

Supplemental material 2: Regression models and thresholds for the optimal model per time segment (DOCX 15 kb)
