# Supplementary material for: Improving Prediction of Favourable Outcome After 6 Months in Patients with Severe Traumatic Brain Injury Using Physiological Cerebral Parameters in a Multivariable Logistic Regression Model
Source: Neurocrit Care. 2020 Feb 13;33(2):542–51. doi: 10.1007/s12028-020-00930-6 (PMC7505885; doi:10.1007/s12028-020-00930-6)
Supplement: Supplementary file 1 — Supplemental material 1: Mean physiological value for each parameter per time segment (DOCX 14 kb) [file 12028_2020_930_MOESM1_ESM.docx]

**Supplemental material 1**

**Table S1.** **Average of the CRASH risk score and the mean of physiological values for each time segment**

|  |  | **Mean (SD)** | | | |
| --- | --- | --- | --- | --- | --- |
|  |  | **0h-6h (n=45)** | **0h-12h (n=45)** | **0h-18h (n=44)** | **0h-24h (n=41)** |
|  | **CRASH risk score (%)** | 58.9 (21.9) | 58.9 (21.9) | 59.4 (21.9) | 60.7 (21.4) |
| **Mean** | **ICP (mmHg)** | 11.1 (5.4) | 11.6 (5.4) | 12.2 (5.7) | 12.4 (5.8) |
|  | **ABP (mmHg)** | 81.0 (7.6) | 80.6 (7.0) | 81.2 (7.1) | 81.1 (6.6) |
|  | **HR (BPM)** | 78.2 (18.2) | 77.8 (17.1) | 77.2 (16.7) | 78.2 (16.0) |
|  | **RAP** | 0.57 (0.26) | 0.61 (0.22) | 0.65 (0.20) | 0.66 (0.19) |
|  | **PRx** | 0.13 (0.24) | 0.11 (0.23) | 0.09 (0.24) | 0.06 (0.23) |
|  | **RAC** | - 0.21 (0.29) | - 0.25 (0.28) | - 0.28 (0.29) | - 0.29 (0.28) |
|  | **PAx** | 0.00 (0.25) | - 0.01 (0.25) | - 0.02 (0.26) | - 0.03 (0.26) |
| **Impairement** | **PRx** | 0.07 (0.08) | 0.07 (0.07) | 0.07 (0.06) | 0.06 (0.06) |
|  | **PAx** | 0.07 (0.09) | 0.07 (0.08) | 0.07 (0.08) | 0.07 (0.08) |
|  | **RAC** | 0.11 (0.12) | 0.10 (0.10) | 0.10 (0.10) | 0.10 (0.10) |
|  | **ICP** | 0.14 (0.44) | 0.22 (0.76) | 0.33 (1.24) | 0.40 (1.58) |
| **Slope** | **PRx (PRx/min)** | - 0.18 (1.20) | - 0.16 (0.71) | - 0.20 (0.47) | - 0.20 (0.39) |
|  | **RAC (RAC/min)** | - 0.61 (1.31) | - 0.29 (0.65) | - 0.25 (0.48) | - 0.22 (0.36) |
|  | **PAx (PAx/min)** | - 0.40 (1.17) | - 0.12 (0.55) | - 0.12 (0.35) | - 0.14 (0.30) |

***Table legend.*** *Average of the CRASH risk score and the mean of physiological values for each time segment. Noticeable is that the mean of cerebral autoregulatory parameters changes with different time periods and that the slope of RAC and PAx differs strongly in the 0h-6h time period compared to the rest of the time periods.*
