# Supplementary material for: Improving Prediction of Favourable Outcome After 6 Months in Patients with Severe Traumatic Brain Injury Using Physiological Cerebral Parameters in a Multivariable Logistic Regression Model
Source: Neurocrit Care. 2020 Feb 13;33(2):542–51. doi: 10.1007/s12028-020-00930-6 (PMC7505885; doi:10.1007/s12028-020-00930-6)
Supplement: Supplementary file 2 — Supplemental material 2: Regression models and thresholds for the optimal model per time segment (DOCX 15 kb) [file 12028_2020_930_MOESM2_ESM.docx]

**Supplemental material 2**

To predict new patients as favourable or unfavourable, calculate the Y value as displayed in Equation 1 using the corresponding regression coefficients given in Table 4-8 and compare Y to the threshold given. If Y is equal to or higher than the corresponding threshold, the patient is predicted to have an unfavourable outcome. Otherwise, the patient is predicted to have a favourable outcome.

**Equation 1. Prediction of new patients**

$$Y=Intercept+\beta_{1}*Parameter 1+\beta_{2}*Parameter 2+\ldots+\beta_{n}*Parameter n$$

**Equation legend.** *Calculation of the value which can be compared to the threshold to predict unfavourable outcome in new patients. Β stands for the regression coefficient, where parameter stands for the value measured for the corresponding parameter. N stands for the number of parameters included in the model.*

**Table S2. Regression coefficients and threshold for the 0h-6h model.**

| **Model 0h-6h** | **Threshold** |
| --- | --- |
|  | 1.259 |
|  | **Regression coëfficiënt** |
| **Intercept** | 19.3 |
| **CRASH risk score** | 0.161 |
| **Mean ABP** | -0.375 |
| **Slope of PRx** | 2.13 |
| **Slope of RAC** | 2.46 |
| **Slope of PAx** | - 4.96 |

***Table legend.*** *Regression coefficients and threshold for the 0h-6h model. An increase in CRASH, slope of PRx and slope of RAC corresponds to an higher probability of unfavourable outcome. An increase ~~decrease~~ in the mean ABP and slope of PAx corresponds to a lower probability of unfavourable outcome.*

**Table S3. Regression coefficients and threshold for the 0h-12h model.**

| **Model 0h-12h** | **Threshold** |
| --- | --- |
|  | 1.166 |
|  | **Regression coëfficiënt** |
| **Intercept** | 2.78 |
| **CRASH risk score** | 0.083 |
| **Mean ABP** | -0.11 |
| **PRx impairement** | 15.2 |

***Table legend.*** *Regression coefficients and threshold for the 0h-12h model. An increase in CRASH and PRx impairment corresponds to an higher probability of unfavourable outcome. An increase ~~decrease~~ in the mean ABP corresponds to a lower probability of unfavourable outcome.*

**Table S4. Regression coefficients and threshold for the 0h-18h model.**

| **Model 0h-18h** | **Threshold** |
| --- | --- |
|  | 0.136 |
|  | **Regression coefficient** |
| **Intercept** | -6.96 |
| **CRASH risk score** | 0.166 |
| **Mean ABP** | -0.113 |
| **Mean PRx** | 19.2 |
| **Mean PAx** | -25.0 |
| **Slope of RAC** | 1.94 |
| **RAC impairment** | 44.4 |

***Table legend.*** *Regression coefficients and threshold for the 0h-18h model. An increase in CRASH, mean PRx, slope of RAC and RAC impairment corresponds to an higher probability of unfavourable outcome. An increase ~~decrease~~ in the mean ABP and mean PAx corresponds to a lower probability of unfavourable outcome.*

**Table S5. Regression coefficients and threshold for the 0h-24h model**

| **Model 0h-24h** | **Threshold** |
| --- | --- |
|  | -1.506 |
|  | **Regression coëfficiënt** |
| **Intercept** | 4.66 |
| **CRASH risk score** | 0.0885 |
| **Mean ABP** | -0.140 |
| **Slope of PRx** | -4.16 |
| **Slope of PAx** | 8.24 |
| **PRx impairement** | 25.2 |

***Table legend.*** *Regression coefficients and threshold for the 0h-24h model. An increase in CRASH, slope of PAx and PRx impairment corresponds to an higher probability of unfavourable outcome. An increase ~~decrease~~ in the mean ABP and slope of PRx corresponds to a lower probability of unfavourable outcome.*
